# Nobiletin suppresses cholangiocarcinoma proliferation via inhibiting GSK3β

**DOI:** 10.7150/ijbs.78345

**Published:** 2022-09-11

**Authors:** Liping You, Jiacheng Lin, Zhuo Yu, Yihan Qian, Yuting Bi, Fang Wang, Lei Zhang, Chao Zheng, Jinghao Zhang, Wenxuan Li, Yaxuan Cai, Yueqiu Gao, Xiaoni Kong, Xuehua Sun

**Affiliations:** 1 Department of Liver Diseases, ShuGuang Hospital Affiliated to Shanghai University of Chinese Traditional Medicine, Shanghai, China.; 2 Central Laboratory, ShuGuang Hospital Affiliated to Shanghai University of Chinese Traditional Medicine, Shanghai, China.; 3 Shanghai experimental school, Shanghai, China.

**Keywords:** Nobiletin, Cholangiocarcinoma, GSK3β, β-catenin, natural medicine

## Abstract

**Background:** Cholangiocarcinoma (CCA) is a type of hepatobiliary cancer characterized by uncontrolled cell proliferation, with a poor prognosis and high mortality. Nobiletin (NBT) is a promising anti-tumor compound derived from the peels of oranges and other citrus plants, citrus plant. But the effect of NBT on CCA remains unknown.

**Results:** Our data showed that NBT suppressed CCA cell proliferation *in vitro* and *in vivo*. Colony formation and Edu assay indicated that NBT inhibited cell proliferation. Cell cycle analysis showed that NBT arrested the cell cycle in G0/G1 phase. Target prediction showed that GSK3β was a direct target. Western blot and immunofluorescence confirmed that NBT reduced the phosphorylation of GSK3β. The antiproliferative effect of NBT was intercepted in GSK3β knockdown CCA cells. The cellular thermal shift assay (CETSA) showed NBT directly bound to GSK3β. Finally, NBT showed an anti-proliferative effect in tumor-bearing mice with no hepatotoxicity.

**Conclusion:** NBT could inhibit CCA proliferation, and the pharmacological activity of NBT in CCA was attributed to its direct binding to GSK3β. We suggested that NBT might be a potential natural medicine in CCA treatment.

## Introduction

Cholangiocarcinoma (CCA) is the second most common primary liver tumor worldwide and has a significantly higher incidence in Southeast Asian countries 1,2, with increasing overall morbidity and mortality 3. Liver flukes, liver cirrhosis, and viral hepatitis are all risk factors for CCA 4. CCA is a slowly progressing cancer arising from bile duct epithelial cells but the outcome is very poor. Because of the difficulty of early diagnosis, many patients receive treatment only when cancer has proceeded to the ending phase. Surgery is the only cure for CCA. Whereas it is considered only for patients with early-stage disease, and local recurrence usually occurs after complete surgical resection, with a low 5-year survival rate 5-6. Therefore, it is necessary to identify a potential therapeutic regimen for CCA.

Nobiletin (5,6,7,8,3,4-hexamethoxyflavone; NBT) is a major component of citrus fruits, particularly orange peels. NBT has exhibited anti-tumor activity in multiple cancer cell types, including renal carcinoma cells 7, breast cancer 8, glioma 9, nasopharyngeal carcinoma 10, liver cancer 11, and gastric carcinoma 12. NBT has been reported to inhibit several hallmarks of cancer pathophysiology, including arresting the cell cycle 13, inhibiting tumor proliferation 14, and accelerating apoptosis 15. It has been considered a potential natural medicine with outstanding pharmacological activity. However, the anti-cancer activity and the mechanism of NBT in CCA had not been reported.

Glycogen synthase kinase-3 (GSK-3) is a serine-threonine kinase controlling cell division, proliferation, and survival. GSK3β can phosphorylate pro-cancer molecules and has been recognized as a potential anti-tumor target 16. Aberrant GSK-3β activity is relevant to multiple tumor-related diseases, and it is prominent as a potential therapeutic target 17-18. Some GSK-3β inhibitors, alone or in combination with chemotherapy drugs, are used in preclinical studies 19. Wnt/β-catenin is a highly conserved signaling pathway in evolution, including β-catenin and GSK-3β. Wnt/β-catenin signaling is involved in a wide variety of human cancers 20. Abnormally activated Wnt/β-catenin signaling could inactivate GSK-3β to inhibit β-catenin degradation 21-22. Aberrant accumulated β-catenin would translocate to the nucleus and induce the transcription of many oncogenes such as Cyclin-D1-binding protein 1(Cyclin D1) and Cyclin-dependent kinase 4(CDK4), thus promoting tumor progression 23-24.

In the study, we observed the inhibitory effect of NBT on CCA. We detected the function of NBT on tumor proliferation and the cell cycle of CCA and used computational pharmacology methods to explore the mechanism of NBT, which was verified by siRNA and the CETSA. We finally detected the anti-tumor effect and safety of NBT on tumor-bearing mice.

## Methods

### Drugs

Nobiletin (NBT, purity ≥98%, purchased from MCE (HY-N0155) was dissolved in dimethyl sulfoxide (DMSO) at a concentration of 100 mmol, and NBT was storage at -80 °C.

### Cell isolation and culture

Primary hepatocyte was isolated using C57BL/6J mice, in short, the liver was poured into EGTA buffer (Sangon Biotech, A600077-0025) through the portal vein for 8 min. Then perfusion buffer containing collagenase (Sigma, V900893-1G) was infused for 10 minutes. The isolated liver was moved out through a 70-μm cell strainer, and the hepatocyte was obtained by centrifugation at 400 rpm for 5 minutes. Human extrahepatic bile duct carcinoma cell line TFK1 and human cholangiocarcinoma cell line RBE were purchased from the Cell Bank of the Chinese Academy of Sciences (Shanghai, China). Primary hepatocyte and TFK1 were cultured in DMEM medium (Gibco, C11885500). RBE were cultured in RPMI 1640 medium (Gibco, C11875500CP). All mediums were supplemented with 10% fetal bovine serum (Gibco, 10099141C), Penicillin 100 U/mL, and Streptomycin 100 μg/mL (Gibco, 15140122) at 37 °C and 5% CO_2_.

### Cell vitality assay

Cell vitality was determined using the Cell Counting Kit-8 assay (CCK-8) (Dojindo, CK04). 100 μL cell suspensions (5000 cells/well) were plated in 96-well plates. Different concentrations of NBT (0, 1, 6.25, 12.5, 25, 50, and 100 μmol/mL) and the CCA cells were incubated for 12, 24 and 48 hours (6 wells per concentration). 10 μL CCK-8 reagents were added to incubation for 2 hours. The OD values were measured at the wavelength of 450 nm by the multi-mode microplate reader (SpectraMax iD5, USA). The above tests are performed 3 times independently.

### Cell apoptosis assay

Tumor cell apoptosis was tested by the Annexin V-FITC Apoptosis Detection Kit I (BD, USA, 556547). Briefly, 2 mL cell suspensions (1×10^5^ cells/well) were cultured in 6-well dishes. The NBT (0, 50, and 100 μmol/mL) combined with the cells were incubated for 48 hours, and then separated from the culture plate using tyrisin. Cell suspensions were washed by phosphate-buffered saline (PBS) and resuspended to be stained with FITC Annexin V reagent and Propidium Iodide (PI) was used to measure apoptosis by flow cytometry (BD, FACSCantoTM II). The above tests are performed 3 times independently.

### Colony formation and crystal violet staining

1×10^3^ cells/well were incubated in a fresh 6-well dish with NBT (0, 6.25, 12.5, 25, 50, and 100 μmol/ml) for 14 days. Then the cells were fixed with 4% paraformaldehyde for 30 mins and then stained with 0.1% crystal violet for 15 mins. Finally, the colonies were imaged, and the number of colonies with more than 50 cells was counted. The above tests are performed 3 times independently.

### EdU staining

EdU is a soluble biomarker that is integrated into newly synthesized DNA by cells within a sample for detecting cell proliferation. 1×10^5^ cells/well were seeded and incubated in a fresh 6-well plate. Different concentrations of NBT (0, 50, and 100 μmoL/L) were then added, and cells were incubated for 24 or 48 hours. Using the BeyoClickEdU Cell Proliferation Kit with Alexa Fluor 488 (Beyotime, C0075S, Jiangsu, China), Hoechst 33342 was used for nuclear staining, then the cells were imaged by the inverted fluorescence microscope (Nikon, TS100-F, Japan). The above tests are performed 3 times independently.

### Cell cycle assay

The TFK1 and RBE were grown to approximately 70% confluence and treated with NBT (0, 50, and 100 μm) for 24 hours. The cell cycle was measured with the Cell Cycle Analysis Kit (Beyotime, C1052, Jiangsu, China). Briefly, 24 hours after treatment, the cells were washed twice with cold PBS, centrifuged, and fixed in 70% ethanol at 4 °C for 24 hours. They were then washed with PBS, incubated with RNase at 37 °C for 30 min, stained with propidium iodide, and analyzed by flow cytometry. The above tests are performed 3 times independently.

### Western blot

Samples were lysed with RIPA (Thermo Scientific, 89900) containing protease inhibitor (MCE, HY-K0010) for 30 mins, then centrifugation at 1200 rpm for 10 mins at 4 °C. The Nuclear and Cytoplasmic Extraction Reagents (Thermo Scientific, 78833, USA) were applied to extract nuclear protein from CCA cells. The protein samples were quantified by the BCA. Then western blot was performed with primary and secondary antibodies. See Table 1 for primary antibodies.

### RT-qPCR

Total RNA extraction Kit (Bioteke Corporation, RP4002) extracts total RNA. Reverse transcription of 500 ng RNAs using HiScript II Q RT SuperMix for qPCR kits (Vazyme, R222-01). Dilute cDNA with ChamQ SYBR qPCR Master Mix (Vazyme, Q311-02) and add gene-specific primers for qPCR. The relative expression of target mRNA was normalized by β-actin. See Table 2 for primer sequences.

### RNA interference

Transient knockdown of GSK3β and JNK1 was achieved in TFK1 and RBE cells using siRNA-GSK3β (sequence-CUCAAGAACUGUCAAGUAATT; s6239; Invitrogen) and siRNA-JNK1 (sequence-GCCGACCAUUUCAGAAUCATT; GenePharma). Irrelevant control siRNA was used for control. Transfection was carried out by Lipofectamine™ 3000 Transfection reagent (Invitrogen, L3000001, Carlsbad, CA). 48 hours after transfection, the expression of genes was observed by western blot and RT-PCR.

### Target prediction and molecular docking

To predict the target of NBT, the webserver PharmMapper (http://lilab-ecust.cn/pharmmapper/) was used 25. The potential targets of CCA and NBT were acquired from Genecards (https://www.genecards.org/) and TCMSP (https://tcmsp-e.com/). For molecular docking, the molecular structure of NBT and GSK3β were acquired from PubChem (https://pubchem.ncbi.nlm.nih.gov/) and PROTEIN DATA BANK (https://www.rcsb.org/).To test the binding affinity of NBT on GSK3β, we used the molecule visual software Chimera 1.16 and Auto Dock VINA. Automatic docking VINZA is a set of automatic docking tools. It is widely used to predict small molecules, such as drug candidates. The protein structure of GSK3β (5HLP) was acquired on the online web of RCSB PDB and NBT was acquired on PubChem. The docking score is based on the Auto Dock VINA score.2D visualization of docking pose is using visual software Discovery Studio 2020.

### Cellular Thermal Shift Assay (CETSA)

As previously described 26-27, briefly, cells were cultured for 1 day with fresh medium on the 10-cm plate. On the test day, cells were exposed to the indicated concentration of compounds (200 μM NBT) for the specified time (120 min). The control group was incubated with an equal volume of DMSO. Following incubation, the cells were washed with PBS to remove excess NBT/DMSO. The suspension was centrifuged at 340 g and 25 °C for 5 min, washed twice with PBS, and diluted with PBS to 100 million cells/mL. After which they were subjected to a 3-minute heat shock to the appropriate heat cycle (42 to 62 °C) for generating melt curves followed by rapid cooling to 25 °C. Then the cells were lysed in liquid nitrogen for 3 min through three freeze-thaw cycles. Centrifuge the pelleted proteins and cell debris at 11,800 g for 20 min to make pellets. Transfer the supernatant to gel loading buffer and analyze protein levels with western blot.

### Immunofluorescence staining

TFK1 and RBE cells (1×10^5^ cells/well) were incubated in the 12-well plate, and the concentrations of NBT (50 μmoL/L) combined with cells were cultured for 24 hours, then fixed with 4% paraformaldehyde. After 0.1% Triton X-100 breaks the cell membrane, samples are blocked with 10% BSA. Active β-catenin diluted in 1% BSA was added and incubated overnight. After repeated washes with PBS, the samples were incubated with a rabbit-anti-human antibody diluted in 1% BSA (1:200). Finally, the cells were stained with DAPI (Meilunbio, MA0222, China) dyeing solution, then the cells were imaged by the inverted fluorescence microscope (Nikon, TS100-F, Japan).

### Xenograft nude mice model

A xenograft nude mice model was applied to research. Male nude mice aged 4-5 weeks were obtained from Shanghai Jiesijie Laboratory Animal Co., Ltd. The 2 × 10^6^ TFK1 cells were resuspended in 50 μL PBS, mixed with 50 μl Matrigel (BD Biosciences, MA, USA), and implanted subcutaneously into the right armpit of mice. After 12 days of inoculation when the tumors grew to 100 mm^3^, mice were randomly assigned to five groups, including control, NBT low dose (NBT-L), NBT high dose (NBT-H), cisplatin (positive control), and combined treatment (NBT-L+ cisplatin) groups. The mice in NBT-L and NBT-H groups were intraperitoneally injected with NBT at 25 mg/kg/day and 50 mg/kg/day respectively. Cisplatin was intraperitoneally injected at 4 mg/kg/week. Combined treatment of cisplatin (4 mg/kg/week) and NBT (25 mg/kg/day) was conducted to determine the synergistic effects. (NBT purity ≥95%, purchased from Shanghai Yuanye Bio-Technology Co., Ltd, S31600, NBT was dissolved in the 10% DMSO+40% PEG300+5% Tween-80+45% saline; cisplatin was dissolved in the saline water). Tumors were measured once every 3 days and calculated by the equation: Volume = 0.5 × Length × Width^2^. After 3 weeks of treatment, the mice were sacrificed by cervical dislocation. The tests about animals were approved and performed by the Animal Care and Use Committee of Shanghai University of Traditional Chinese medicine.

### Immunohistochemistry

Tumor samples were fixed with 4% paraformaldehyde, all specimens were sliced into 5-µm sections. Perform antigen extraction with sodium citrate buffer. After 1 h incubation with 10% serum, incubated overnight with primary antibodies at 4 °C. The subsequent DAB-staining (Beyotime, P0203; China) procedures were performed by the manufacturer's process.

### TUNEL Assay

The TUNEL assay was applied to examine apoptosis cells of cancer. A TUNEL kit (Beyotime, C1098, Jiangsu, China) was performed by the specification.

### Serum biochemical test

The serum ALT concentration was assessed by Alanine aminotransferase Assay Kit (Nanjing Jiancheng Bioengineering Institute; C009-2-1; China) according to the manufacturer's protocol.

### Histological staining

The tissue of the lung, liver, spleen, and kidney should be treated with 4% paraformaldehyde for at least 24 hours. Paraffin embedding and securing was adopted to ensure the thickness of the tissue section was 5 µm. All samples were stained with hematoxylin and eosin (H&E) solution by the standardization procedure.

### Statistical analysis

The Graphpad Prism 8 software was used for all statistical analyses. Results were described as means ± standard deviation. Paired and unpaired data were evaluated using the Student *t*-test. Multi-group comparison was evaluated using one-way AONVA. *p*< 0.05 was considered to be statistically significant (*p<0.05; **p<0.01; ***p<0.001).

## Results

### Nobiletin suppressed human cholangiocarcinoma TFK1 and RBE cell proliferation

**Figure 1:**
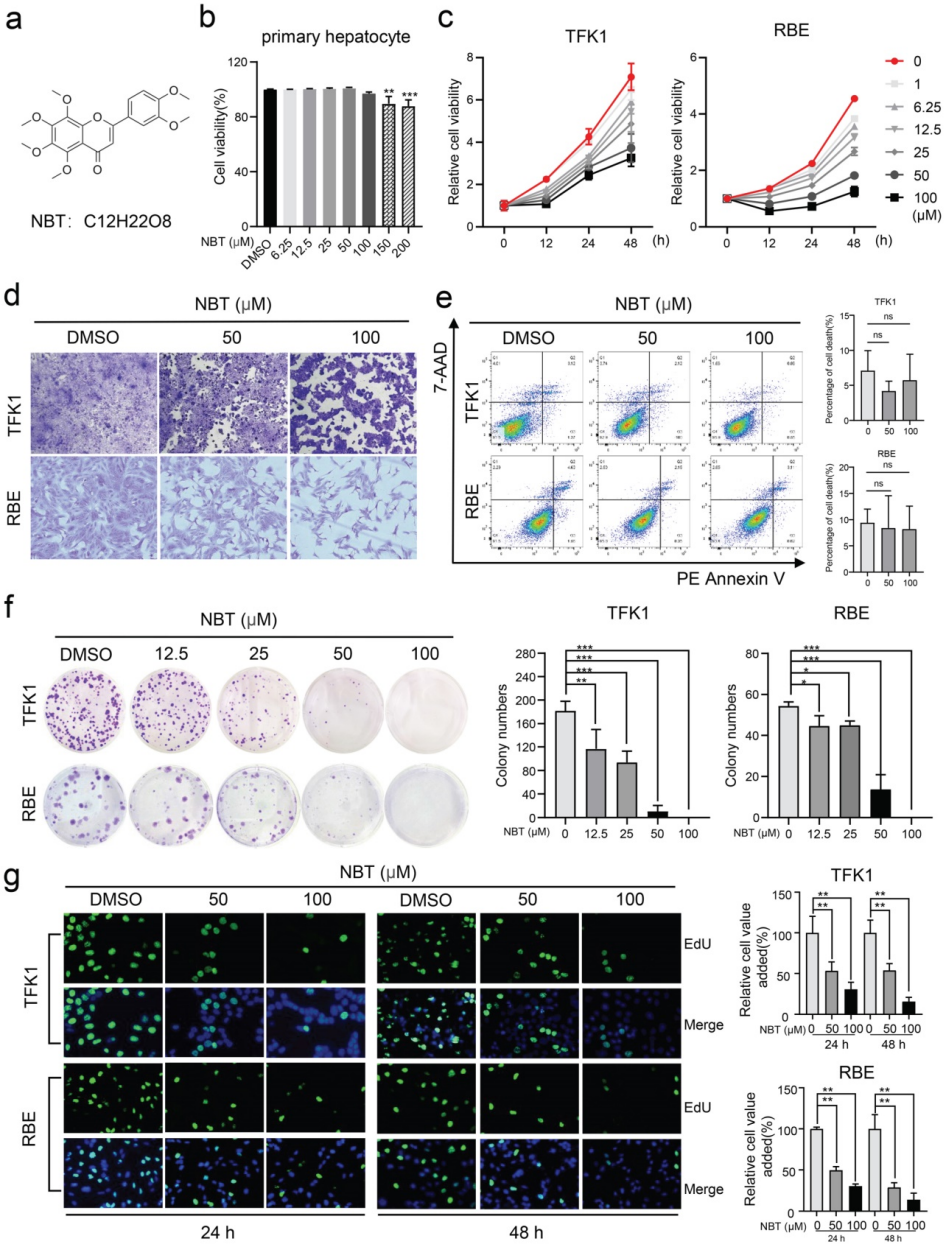
** NBT inhibited CCA cells proliferation. (a)** The chemical structure of NBT. **(b)** The viability of primary hepatocyte was assessed after treatment with different doses of NBT for 48h. **(c)** The viability of TFK1 cells and RBE cells was assessed after treatment with different doses of NBT and at different times. **(d)** Representative cell morphological changes. **(e)** Representative results of annexin V/FITC/PI staining and quantitative analysis, *p< 0.05, **p< 0.01. **(f)** Monolayer culture; quantitative analyses of colony numbers are shown, *p< 0.05, **p< 0.01. **(g)** Representative results of EdU staining and quantitative analyses, *p< 0.05, **p< 0.01.

The CCK-8 assay showed that NBT (Fig.1a) has no toxic effect on primary hepatocytes when the concentration is below 100μM (Fig.1b). To test the bioeffects of NBT in CCA, TFK1 and RBE cell lines were treated with NBT *in vitro*. The CCK-8 result suggested that NBT increased cell viability of TFK1 and RBE cell lines dose-dependently and time-dependently (Fig. 1c). Moreover, representative cell morphological changes after crystal violet staining indicated reduced density of CCA cells after NBT treatment (Fig. 1d). To determine whether NBT can induce cellular death, we performed an Annexin V-FITC dual staining. The data of flow cytometry did not show any increase of dead cells in TFK1 and RBE cells with NBT treatment (Fig. 1e). Further, to state the antiproliferative effect of NBT instead, we utilized a colony formation assay and EdU staining. As the result, NBT notably suppressed the colony formation (Fig. 1f-g), which suggests that NBT treatment inhibited the CCA cell proliferation.

### Nobiletin induced cell cycle arrest in G0/G1 phase

**Figure 2:**
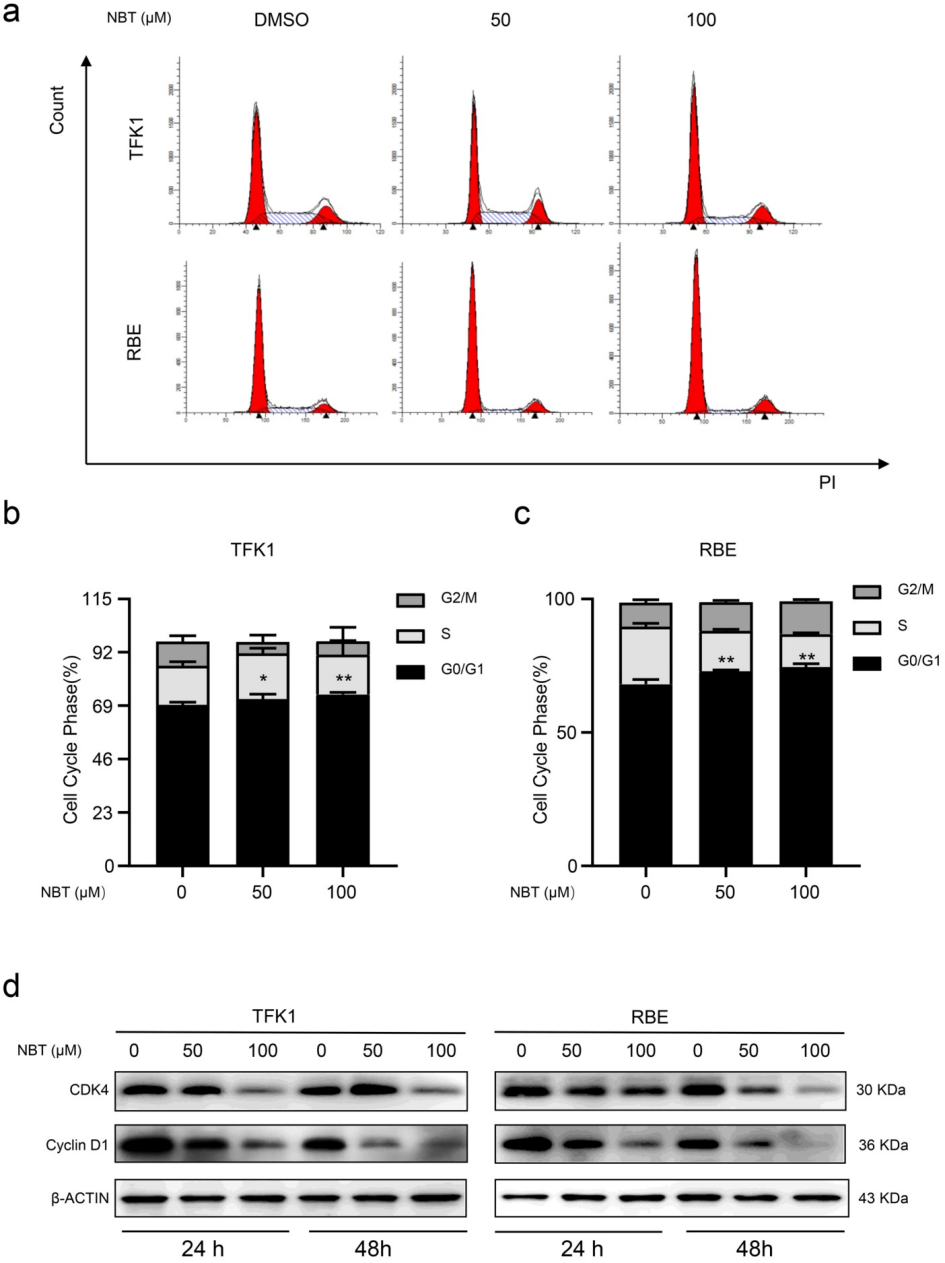
** NBT induced CCA cells cycle arrest in G0/S1. (a)** Representative results of cell cycle and quantitative analyses after 48 hours of NBT treatment. **(b, c),** *p< 0.05, **p< 0.01. **(D)** The expression of G0/G1 cell cycle signal regulators, Cyclin D1 and CDK4 were examined by western blot after NBT treatment for 24 hours and 48 hours.

To explore how NBT inhibited cell proliferation by affecting the cell cycle, we performed flow cytometry. The result showed that NBT promoted G0/G1 phase arrest, with fewer cells in the S phase (Fig. 2a-c). To further observe the NBT induced cell cycle arrestment in the TFK1 and RBE cells, we performed western blotting on the proteins involved in the G0/G1 phase. As a result, it was Cyclin D1 and CDK4 expression levels were downregulated during the G0/G1 arrestment in the cancer cells (Fig. 2d). All the results indicated that NBT treatment effectively decreases tumor proliferation by inducing G0/G1-phase arrest.

### Prediction and validation of nobiletin suppression CCA cell growth

**Figure 3:**
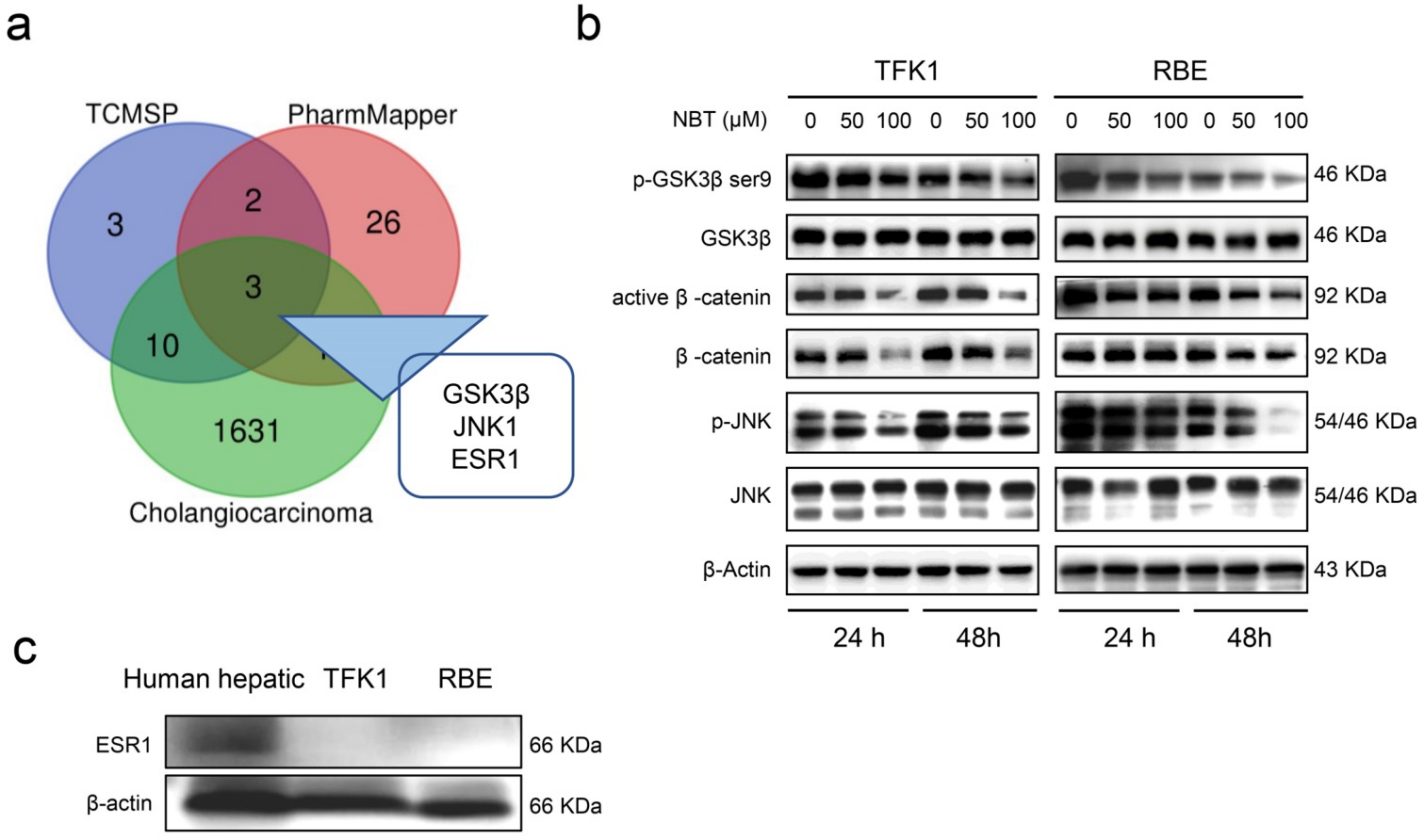
** Prediction and validation of NBT suppression CCA cell growth. (a)** The intersection of target proteins of NBT and CCA. **(b)** Representative results of p-GSK3β, GSK3β, active β-catenin, β-catenin, p-JNK1, and JNK1 protein levels by Western blot analysis after TFK1 and RBE cells were treated with NBT (0, 50, 100 µM) for 24 hours and 48 hours. **(c)** Protein levels of ESR1 by Western blot analysis.

Computational biology and system pharmacology have been shown to contribute to exploring pharmacological targets of compounds. Drug target genes of NBT were searched through TCMSP and PharmMapper databases, and CCA-related target genes were searched through the GeneCards database. The intersection of target genes of NBT and CCA was processed, with 3 intersection genes found, namely Glycogen synthase kinase-3 beta (GSK3β), Mitogen-activated protein kinase 8 (JNK1), and Estrogen receptorα (ESR1) (Fig. 3a). Western blot showed that NBT greatly diminished p-GSK3β^Ser9^ and p-JNK1 protein levels, but not total GSK3β and total JNK1 protein levels. Moreover, the downstream protein of GSK3β, including β-catenin and active β-catenin were also down-regulated (Fig. 3b). However, ESR1 was not expressed in TFK1 and RBE cells (Fig. 3c).

### Nobiletin suppressed CCA cell growth by inhibiting GSK3β signaling pathways in TFK1 and RBE cells

**Figure 4:**
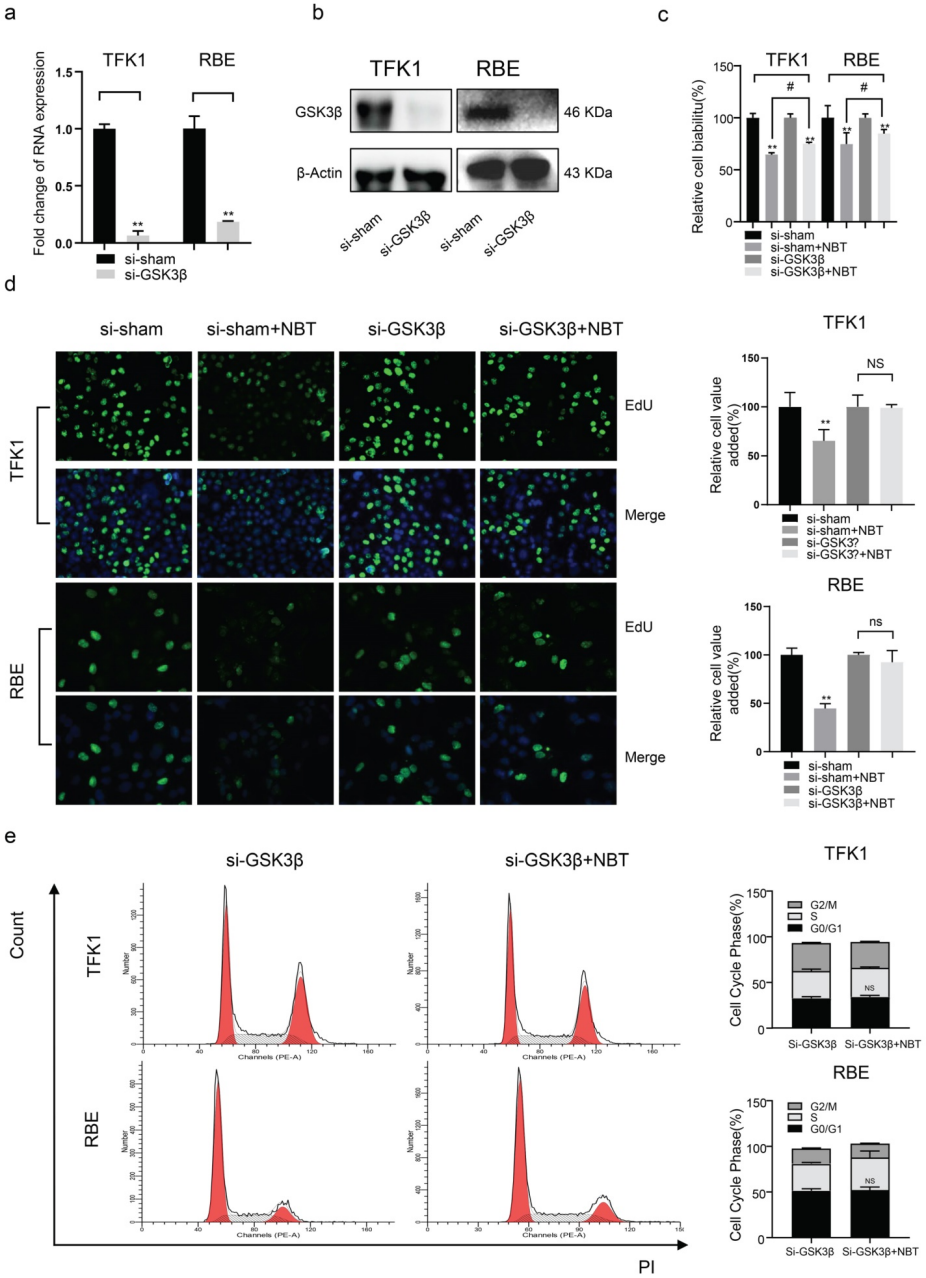
** The biological function of tumor cells was significantly inhibited after GSK3β knockdown. (a)** GSK3β mRNA levels of tumor cells after GSK3β knockdown by siRNA. **(b)** GSK3β protein levels of tumor cells after GSK3β knockdown by siRNA. **(c)** The cell viability of tumor cells after GSK3β knockdown by CCK-8 assay, *p< 0.05, **p< 0.01, # *p*< 0.05, ## *p*< 0.01. **(d)** The proliferation level of tumor cells after GSK3β knockdown by EdU staining, *p< 0.05, **p< 0.01. **(e)** Cell cycle analysis after GSK3β knockdown, *p< 0.05, **p< 0.01.

**Figure 5:**
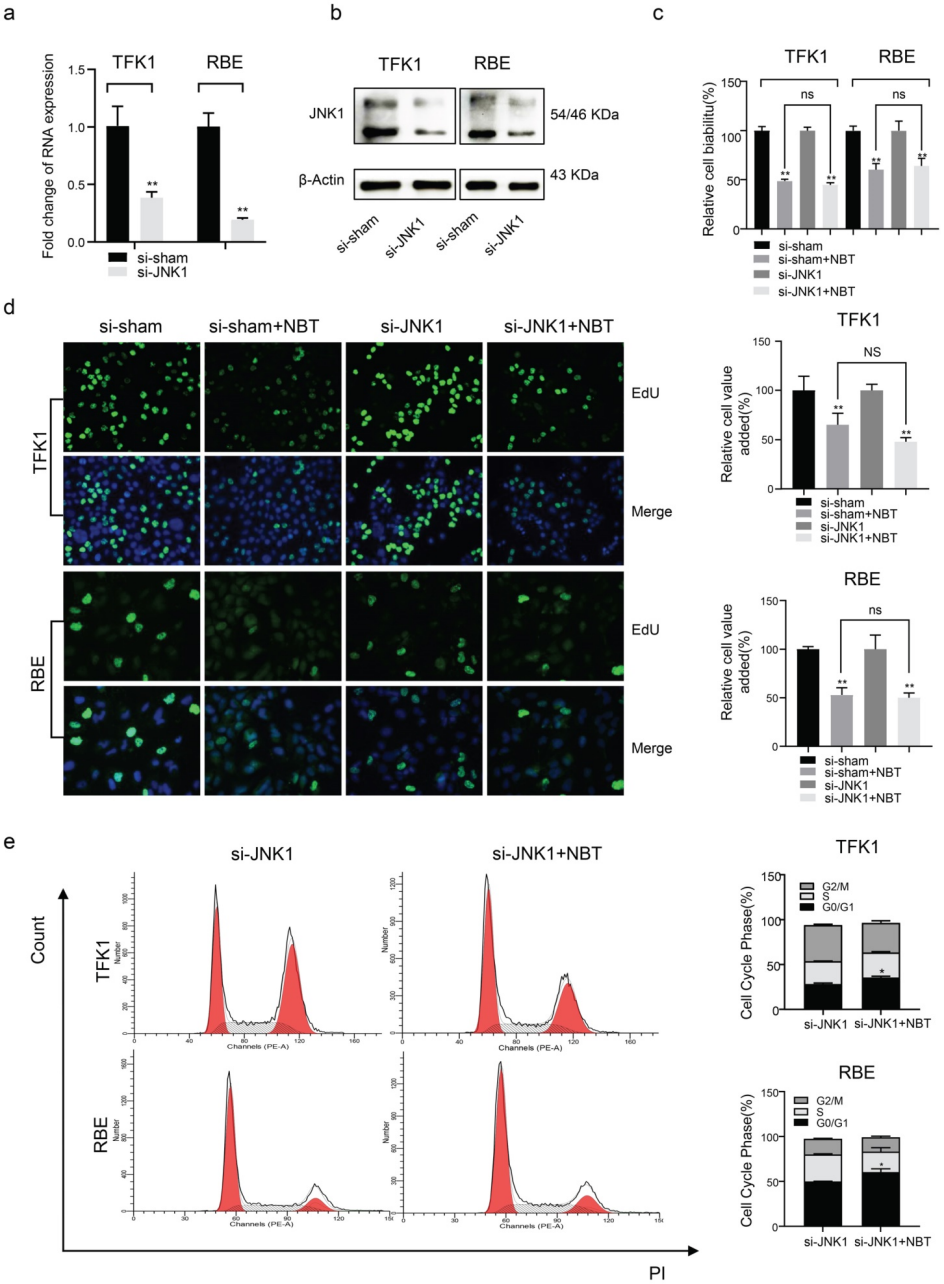
** The biological function of tumor cells remained after JNK1 knockdown. (a)** JNK1 mRNA levels of tumor cells after JNK1 knockdown by siRNA. **(b)** JNK1 protein levels of tumor cells after JNK1 knockdown by siRNA. **(c)** The cell viability of tumor cells after JNK1 knockdown by CCK-8 assay. **(d)** The proliferation level of tumor cells after JNK1 knockdown by EdU staining, *p< 0.05, **p< 0.01. **(e)** Cell cycle analysis after JNK1 knockdown, *p< 0.05, **p< 0.01.

To clarify the role of GSK3 β and JNK1 in the antiproliferative effect of NBT, siRNA was used to deplete GSK-3β and JNK1 expression in TFK1 and RBE cell lines. As the result, western blotting and RT-qPCR indicated that GSK3β and JNK1 significantly decreased protein and mRNA levels (Fig. 4a, b, and Fig 5a, b). Using the CCK-8 vitality assay, we found that in GSK-3β knockdown cells, the ability of NBT to suppress CCA cell activity was significantly weakened (Fig. 4c), whereas this phenomenon did not occur after JNK1 knockdown (Fig. 5c). The EdU staining assay also confirmed these results (Fig. 4d and Fig. 5d). In the end, we performed flow cytometry to observe the function of NBT treatment on the cell cycle in GSK-3β and JNK1 knockdown cells. The data showed that the cell cycle arresting activity of NBT in the TFK1 and RBE cells was significantly weakened (Fig. 4e), while this phenomenon did not occur after JNK1 knockdown (Fig. 5e). These results confirmed that NBT regulated TFK1 and RBE CCA cell viability and proliferation mainly through the GSK3β signaling pathway.

### Nobiletin directly bound to GSK3β to intercept inactivation

**Figure 6:**
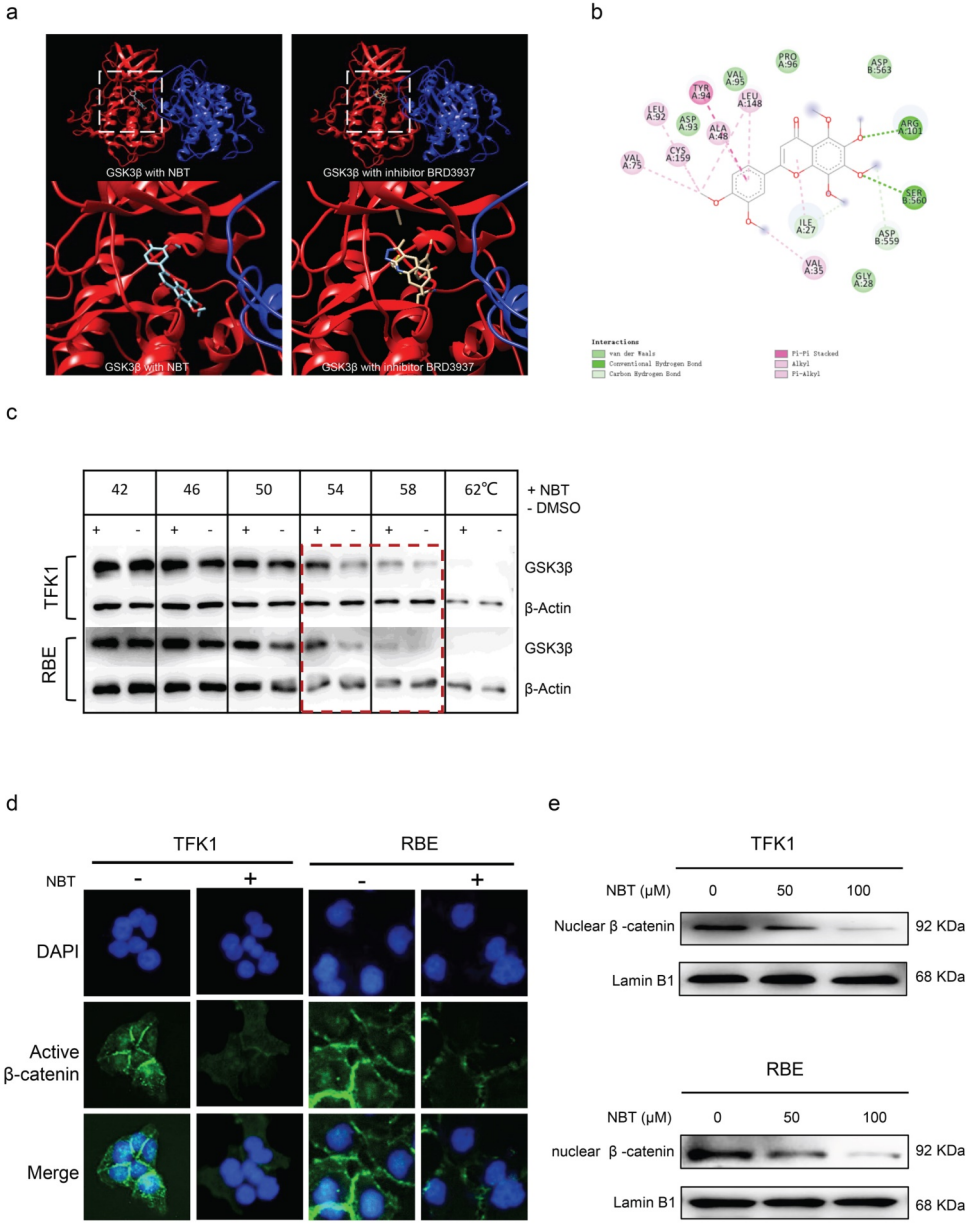
** NBT reduced the nuclear shift of β-catenin by targeting GSK3β. (a, b)** Molecular docking showed the NBT has a high affinity on GSK3β and the binding pose located on the active pocket which could affect the phosphorylation of GSK3β. **(c)** The illustration of the CETSA following quantification of the Western blots. **(d)** Localization of active β-catenin in TFK1 and RBE cells treated with NBT 50 µm for 24 hours or vehicle detected by immunofluorescence staining. **(e)** Protein expression of β-catenin in TFK1 and RBE cells treated with NBT or vehicle determined in nuclear fractions by Western blot. Lamin B1 was used as nuclear protein control.

Molecular docking showed the NBT has a high affinity on GSK3β and the binding pose located on the active pocket which could affect the phosphorylation of GSK3β (similar to GSK3β inhibitor BRD3937) (Fig. 6a). The binding score is -7.5 which implied a stronger affinity (Fig. 6b). Moreover, we verified the direct effect between GSK3β and NBT using CESTA. CESTA is a technique to detect the thermal stability of proteins upon ligand binding. Since the direct bonding of small molecules will enhance or reduce the thermal stability of proteins, CESTA has been used to study the direct pharmacological target of compounds 26,28. The results of CESTA indicated that the GSK3β expression of the NBT-treatment group was remarkable higher than non-NBT treated at 54 °C and 58 °C (Fig. 6c), this result shows that NBT affected the thermo-stability of GSK3β. Finally, through the immunofluorescence of cell and nuclear fractionation, it was found that the β-catenin accumulation decreased after NBT treatment in TFK1 and RBE cell nuclear (Fig. 6d and e), all the results further confirm that NBT regulated the proliferation of CCA through the target of GSK3β.

### Nobiletin inhibited tumor proliferation *in vivo*

**Figure 7:**
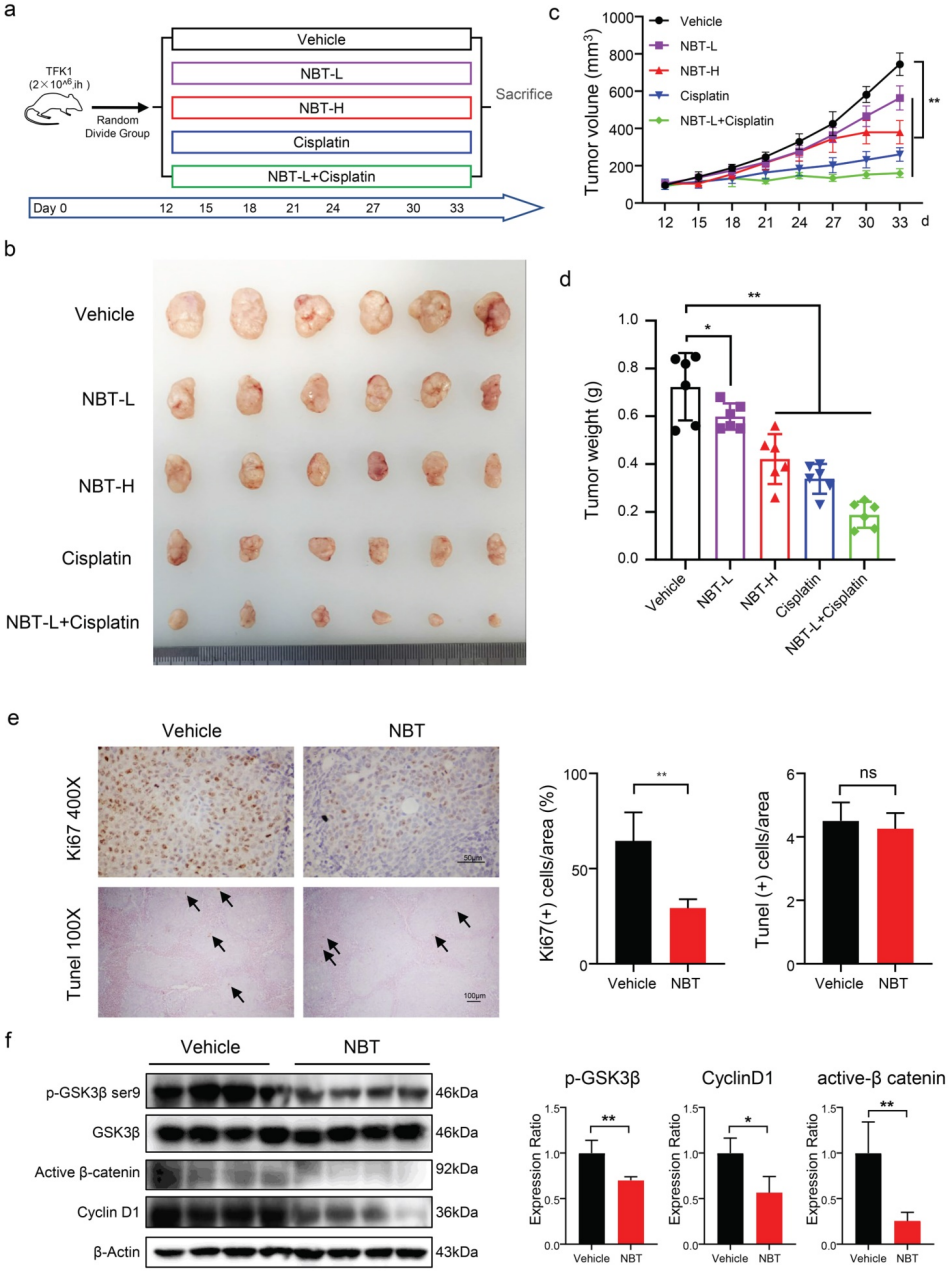
** Anti-tumor function of NBT on CCA *in vivo*. (a)** Scheme of CCA inoculation and systemic injection. **(b)** Representative image of TFK1 xenograft tumors in each group. **(c)** Tumor volume in each group. Data were expressed as the means ± standard deviations (SDs). **(d)** Tumor weight in each group. Data were expressed as the means ± standard deviations (SDs). *p< 0.05, **p< 0.01. **(e)** Immunohistochemical staining of Ki67and TUNEL assay in tumors from the vehicle or NBT-H treated mice and Ki67 quantitative analysis, *p< 0.05, **p< 0.01. **(f)** The expression of p-GSK3β, GSK3β, Cyclin D1 and active β-catenin were detected by western blot.

A xenograft tumor model was established with TFK1 cells subcutaneously injected into BALB/c nude mice to estimate the effect of NBT on CCA *in vivo* (Fig. 7a). The mice in the vehicle group showed rapid tumor growth, while 25mg/kg/d and 50mg/kg/d NBT treatment notably suppressed tumor growth in a dose-dependent manner. The data illustrated cisplatin reduced the average 53% weight of CCA tumors, and 25mg/kg/d and 50mg/kg/d NBT reduced the average 17% and 42% weight of CCA tumors respectively. In addition, combined treatment with a low dose of nobiletin and cisplatin significantly suppressed the tumor growth curve (Fig. 7b, c, and d). Ki67 staining indicated that NBT remarkably inhibited human CCA proliferation, as shown by 2-fold fewer positive tumor cells compared with the counterparts treated by vehicle (Fig. 7e). The TUNEL assay indicated that no obvious positive cells in the vehicle group and NBT-treatment group were been observed, and there was no difference between them (Fig. 7e). These results indicated that NBT treatment inhibited the proliferation of CCA, but did not promote cancer cell apoptosis. Further, western blotting analysis confirmed the result that the NBT induced decrease in tumorigenicity was related to the inhibition of p-GSK3β^Ser9^ and activation of β-catenin and Cyclin D1 (Fig. 7f). This evidence indicated that NBT inhibited xenograft tumor development *in vivo* by suppressing p-GSK3β/β-catenin/Cyclin D1 signaling.

### Nobiletin had no toxicity *in vivo*

**Figure 8:**
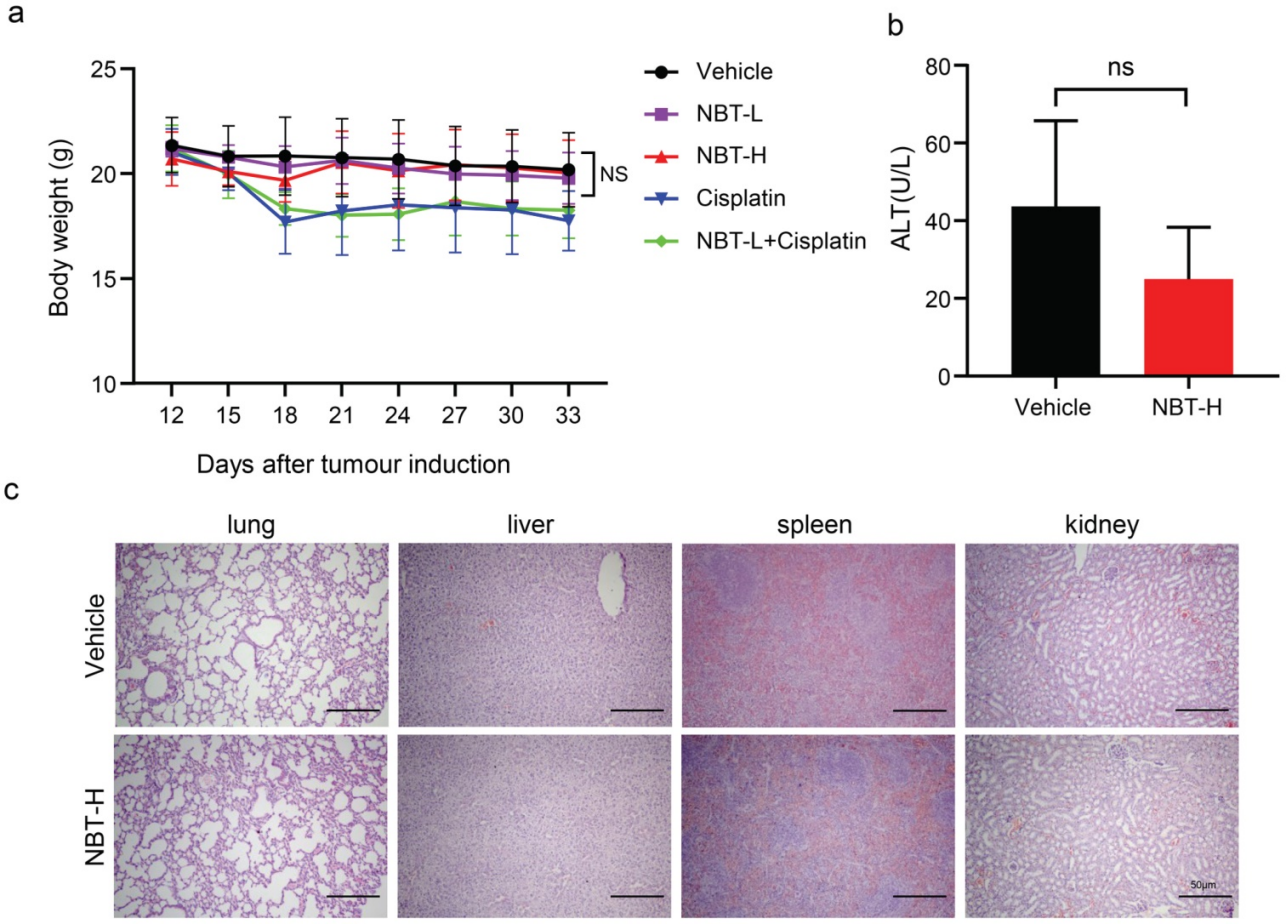
** Bodyweight, ALT, and H&E staining of xenograft tumor sections. (a)** Bodyweight changes between each group. **(b)** ALT changes between control and NBT-H treated mice. **(c)** H&E staining of major organs.

To evaluate the toxic effects of NBT, the changes in body weight and ALT levels in the experimental groups were detected, and various organs were harvested. It is worth mentioning that weight loss occurred in the cisplatin-treated mice, whereas there was no difference in weight between the nobiletin-treated mice and the vehicle mice (Fig. 8a). The serum level of ALT remained within the normal range between the vehicle and NBT-H group (Fig. 8b). There have been no differences of histological phenotypes were found in the vehicle and NBT-H groups, including lung, liver, spleen, and kidney, indicating that NBT had no notable toxicity in the *in vivo* trial (Fig. 8c).

## Discussion

Cholangiocarcinoma (CCA) is usually in an advanced phase when it is diagnosed. Pathological characteristics prolonged biliary injury and abnormal proliferation. CCA arises from the malignant growth of the epithelial lining of the bile ducts. The curative treatment of CCA is still a difficult clinical problem due to it is hard to clarify the diagnosis at the beginning phase and the majority of patients receive treatment when the tumor progress to the development phase.

Some natural compounds with anti-tumor functions could provide a new choice for the CCA remedy. In recent years, researchers have been increasingly interested in exploring potential anticancer compounds from plants. Nobiletin is one of the extensively researched natural compounds, a polymethoxyflavone extracted exclusively from citrus peels, which has diverse good activities including anti-cancer 29, anti-inflammatory 30, antioxidant 31, and anti-aging 32. NBT has been reported to be beneficial to Crohn's disease 33, metabolic syndrome 34, atherosclerosis 35, and Alzheimer's disease 36. The antitumor activity of NBT has attracted attention, however, the anti-cancer activity and the mechanism of NBT in CCA had not been reported.

In recent years, some GSK-3 inhibitors have been developed, some of which are currently undergoing clinical trials. It has been shown that uncontrolled cell growth caused is responsible for the development of cancer. Blocking the cell cycle process is a commonly used anti-cancer method. The cell cycle is a skin-to-biological growth clock that strictly regulates each stage of cell growth, where any mutant or abnormal cells will be stopped at either the G0/G1, S2, or G2/M checkpoints. Regulatory cyclin plays a key role because it requires phosphorylated cyclin-dependent kinase (CDK) complexes to move on to the next phase of the cell cycle.

In this work, we explored the function of NBT in inhibiting the proliferation of CCA. The research results indicated that NBT decreased the proliferation of CCA via targeting GSK3β. After GSK3β bound to NBT, as the subsequent phosphorylation of GSK3β^Ser9^ decreased, with the increase of β-catenin degradation in cytoplasm, the transfer of active β-catenin to the nucleus decreased, which further repressed the expression of Cyclin D1 and CDK4, and finally induced cell cycle arrest in G0/G1 phase. We further demonstrated the *in vivo* anti-tumor activity in a nude mice xenograft tumor model. At a dose of 25 mg/kg/d and 50 mg/kg/d, NBT significantly inhibited the growth of the tumor. In addition, even low NBT (25 mg/kg) combined with cisplatin can could improve the efficacy of cisplatin. Inhibition of p-GSK3 β/β-catenin/Cyclin D1 signaling is the main factor in inhibiting CCA cell growth, which has also been verified *in vivo*. Some reports suggested that the *in vitro* anti-tumor effect of NBT was not achievable *in vivo* due to the low plasma concentration 37-38. However, our study showed that 25 and 50 mg/kg NBT treatment inhibited tumor growth, which may be attributed to the different sensitivity of NBT in different tumor types. The liver is an important organ involved in the metabolism of NBT 38. Bodyweight is often used as a proxy for toxicity and no difference in body weight in NBT-treated mice, indicating that there was no hepatotoxicity of 50 mg/kg NBT in mice. Our *in vitro* experiments showed that NBT had no cytotoxicity, which was consistent with the previous reports 39. The pharmacological activity of NBT on cancer is considered to be more likely to have cytostatic properties rather than cytocidal nature 38, which is consistent with our findings. These findings implied that NBT could be a safer therapy than chemotherapy in terms of controlling the progression and recurrence of CCA.

**Figure 9:**
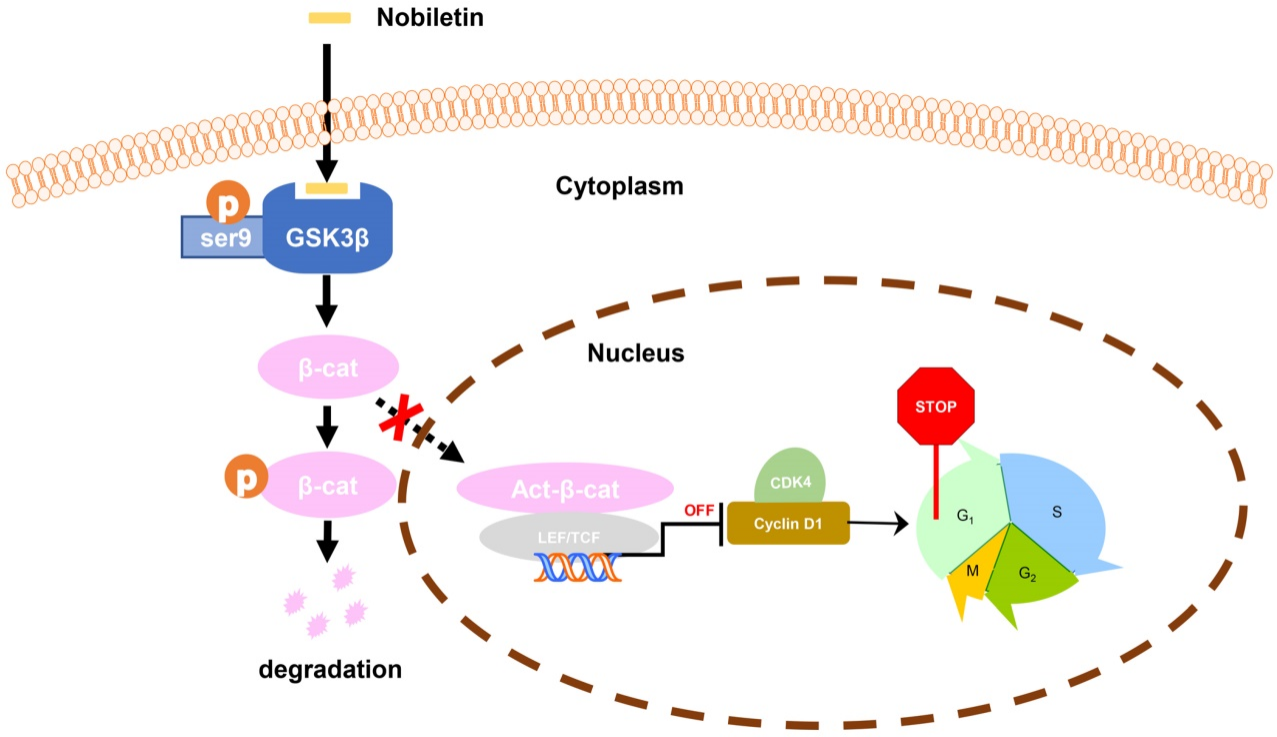
Schematic diagram showing the mechanism of NBT inhibiting proliferation in CCA.

Taken together, we found that the inhibition of p-GSK3β/β-catenin/Cyclin D1 signaling may be the main factor in the growth inhibition of CCA in response to NBT treatment. The study clarified that NBT could be a potential drug for the therapeutic of CCA, highlighting the inhibition of p-GSK3β/β-catenin/Cyclin D1 oncogenic signaling by targeting GSK3β (Fig. 9).

## Conclusion

NBT could inhibit CCA proliferation, and the pharmacological activity of NBT in CCA was attributed to its direct binding to GSK3β. We suggested that NBT might be a potential natural medicine in CCA.

## Figures and Tables

**Table 1:** Antibody used for WB and IHC

Antibody	Source	Manufacturer
Cyclin D1	rabbit	Cell signaling technology; 55506
CDK4	rabbit	Cell signaling technology; 12790
p-GSK3β	rabbit	Cell signaling technology; 9322S
GSK3β	rabbit	Cell signaling technology; 9315S
β-catenin	rabbit	Cell signaling technology; 8480S
Active β-catenin	rabbit	Cell signaling technology; 8814S
Lamin B1	mouse	Abcom; ab16048
p-JNK1	rabbit	Cell signaling technology; 4668S
JNK	rabbit	Cell signaling technology; 9252S
ESR1	mouse	Santa Cruz Biotechnology; sc-8002
β-Actin	rabbit	ABGENT; AP14779b
Ki67	rabbit	Abcom; ab15580

**Table 2:** Primers used for RT-qPCR

Gene	Sequences 5'-3' forward	Sequences 5'-3' reverse	Species
β-actin	CATGTACGTTGCTATCCAGGC	CTCCTTAATGTCACGCACGAT	human
GSK3β	AGGAGAACCCAATGTTTCGTAT	TCCCCTGGAAATATTGGTTGT	human
JNK1	ACACCACAGAAATCCCTAGAAG	CACAGCATCTGATAGAGAAGGT	human

## Abbreviations

GSK3β: Glycogensynthasekinase-3beta
CCA: Cholangiocarcinoma
NBT: Nobiletin
Cyclin D1: Cyclin-D1-binding protein 1
CDK4: Cyclin-dependent kinase 4
JNK1: Mitogen-activated protein kinase 8
ESR1: Estrogen receptor α
CESTA: cellular thermal shift assay
